# Occurrence of *Eimeria* Species Parasites on Small-Scale Commercial Chicken Farms in Africa and Indication of Economic Profitability

**DOI:** 10.1371/journal.pone.0084254

**Published:** 2013-12-31

**Authors:** Kimberly M. Fornace, Emily L. Clark, Sarah E. Macdonald, Boniface Namangala, Esron Karimuribo, Joseph A. Awuni, Olaf Thieme, Damer P. Blake, Jonathan Rushton

**Affiliations:** 1 London School of Hygiene and Tropical Medicine, London, United Kingdom; 2 Royal Veterinary College, North Mymms, Hertfordshire, United Kingdom; 3 Department of Paraclinical Studies, University of Zambia, Faculty of Veterinary Medicine, Lusaka, Zambia; 4 Southern African Centre for Infectious Disease Surveillance, Morogoro, Tanzania; 5 Accra Veterinary Laboratory, Accra, Ghana; 6 Food and Agriculture Organization, Rome, Italy; University of Georgia, United States of America

## Abstract

Small-scale commercial poultry production is emerging as an important form of livestock production in Africa, providing sources of income and animal protein to many poor households, yet the occurrence and impact of coccidiosis on this relatively new production system remains unknown. The primary objective of this study was to examine *Eimeria* parasite occurrence on small-scale commercial poultry farms in Ghana, Tanzania and Zambia. Additionally, farm economic viability was measured by calculating the farm gross margin and enterprise budget. Using these economic measures as global assessments of farm productivity, encompassing the diversity present in regional husbandry systems with a measure of fundamental local relevance, we investigated the detection of specific *Eimeria* species as indicators of farm profitability. Faecal samples and data on production parameters were collected from small-scale (less than 2,000 birds per batch) intensive broiler and layer farms in peri-urban Ghana, Tanzania and Zambia. All seven *Eimeria* species recognised to infect the chicken were detected in each country. Furthermore, two of the three genetic variants (operational taxonomic units) identified previously in Australia have been described outside of Australia for the first time. Detection of the most pathogenic *Eimeria* species associated with decreased farm profitability and may be considered as an indicator of likely farm performance. While a causal link remains to be demonstrated, the presence of highly pathogenic enteric parasites may pose a threat to profitable, sustainable small-scale poultry enterprises in Africa.

## Introduction

Coccidiosis, a host specific intestinal disease caused by *Eimeria* species parasites, has long been recognised as a disease with major economic impact on poultry production within Europe and the developed world. The global cost of coccidiosis to the poultry industry has been estimated to exceed $2 billion per year worldwide [1], [2], [3]. Control relies largely on chemoprophylaxis, but resistance develops rapidly. Live wild-type or attenuated parasite vaccines are available for poultry, although the species-specific nature of immunity induced by exposure to live *Eimeria* incurs a requirement for multiple species and, in some cases, strains for a fully effective anticoccidial vaccine [4]. Seven species of *Eimeria* have been recognised to specifically parasitise the domestic chicken, *Gallus gallus*, each with differing levels of pathogenicity and specific areas of localisation within the digestive tract [4]. Infection with multiple species occurs frequently and clinical outcomes can vary substantially [3], [5], [6]. Following the development of molecular tools for the specific diagnosis of eimerian infection three genetic variants (operational taxonomic units or OTUs) have been described from studies with Australian poultry [7]. Comparative analyses have identified OTU-X as an *Eimeria maxima* population variant, with OTU-Y and OTU-Z defined as cryptic strains or species most closely related to *Eimeria brunetti* and the haemorrhagic *Eimeria tenella*/*Eimeria necatrix* group respectively [7], [8]. The exquisite species-specificity of the immune protection stimulated by eimerian infection raises concerns that existing live vaccines may exert reduced or no control over parasites defined by these cryptic genotypes [7], an important consideration should they become more widely prevalent. Costs associated with coccidiosis include both direct and indirect components; including the cost of control measures, production losses and potential consequences to animal health from resistance to chemoprophylaxis [2], [3]. While coccidiosis has been demonstrated to cause production losses, affecting the profitability of individual farms in Europe and the United States [5], [8], the occurrence and economic relevance of coccidiosis on poultry farms in Africa remains largely unknown.

Within Africa and other areas of the developing world increasing human populations, income levels and urbanisation have led to a rise in demand for livestock products leading to an increase in livestock production, often referred to as the “Livestock Revolution” [9]. Small-scale intensive livestock industries have emerged in peri-urban areas of many developing countries, mainly based on monogastric species with specialised breeds fed on concentrated feeds. With an annual growth rate of 2.1%, poultry is one of the fastest growing livestock sectors, providing an additional source of income and an affordable and accessible source of protein to many poor households [10]. Over 85% of rural households in Sub-Saharan Africa (SSA) keep poultry, with smaller scale commercial farms (less than 2,000 birds per batch) believed to play a critical role in providing households with economic opportunities and ensuring food security [11]. As women frequently manage household poultry production in developing countries, income from poultry can additionally be a method to support women's livelihoods [12]. While traditional (backyard) poultry production systems consist of few free-ranging dual-purpose indigenous birds kept mainly for household use with few inputs and high mortality, small-scale commercial farms raise poultry primarily as an income generating activity, keeping broilers or layers in semi-intensive conditions with food and water usually provided. These emerging small-scale systems have been identified as a target for development and an important source of income for African households, but little is known about the profitability of these enterprises. Adoption of intensive farming methods with limited biosecurity may create an environment conducive to disease. Disease challenges faced by these farms are poorly characterised and have important implications for household poverty and nutritional status. Identification of cost-effective indicators of profitability will be valuable in the development of optimal production systems. Preliminary studies of poultry systems in SSA, primarily based on clinical symptoms and post mortem examinations, indicate coccidiosis is present in backyard and commercial chickens but the species present are largely unknown [13], [14], [15], [16], [17], [18], [19], [20], [21], [22], [23]. The objective of this study was to examine the distribution of *Eimeria* species parasites and investigate the qualitative occurrence of specific species as indicators of profitability for small-scale commercial poultry farms in Ghana, Tanzania and Zambia, as measured by the farm gross margin.

## Materials and Methods

### Ethics statement

The study described was approved following ethical review by the RVC Ethics and Welfare Committee for the use of questionnaires and collection of environmental faecal samples from commercial small-scale poultry production units in Ghana, Tanzania and Zambia. Participants provided verbal informed consent to participate in this study in the absence of a paper-based questionnaire as approved by local ethical review. Environmental faecal samples were collected from private land and small-holdings with the verbal informed consent of the property owner or manager.

### Sample selection and statistical power

The three case study countries for this project were Ghana, Tanzania and Zambia. Small-scale commercial farms raising either broilers or layers were sampled in peri-urban areas of high poultry density (Table 1). Due to differences in availability of information, sampling frames were compiled using different approaches in each of the case study countries using records from veterinary services, poultry suppliers and farmer organizations. In each country all farms identified were compiled into lists of broiler and layer farms. A panel of farms representing each production type in each country was then randomly selected using Microsoft Excel randomizer (Microsoft Corporation, USA). Sample size was selected using WinEpi [24] with the parameters confidence level: 95%, power: 80%, percentage farms estimated to be commercially profitable: 80% and percentage farms estimated to be commercially unprofitable: 20%; indicating a requirement for a minimum sample size of eight farms per system, per country (figures based upon first round observations in Zambia). Thus, ten or more farms were targeted per system, per country, to allow for sample dropout.

**Table 1:** Sampling site locations.

	Ghana	Tanzania	Zambia
Income level*	Lower middle	Low	Lower middle
National poultry population**	44.4 million	33.5 million	35 million
Sampling location	Ga East Muncipal Assembly, Greater Accra	Kibaha Town Council, Greater Dar es Salaam	Greater Lusaka area (within 50 km of Lusaka)
Sampling time	July 2011	April 2011	May 2011

World Bank classifications [35].

FAOStat data for 2010 [36].

### Sample collection

A total of 80 farms were visited during the course of these studies. Faecal samples were collected from 73 farms, excluding seven which were between flocks and could not be included. Farmers from 67 farms consented to be interviewed on production parameters, farm background, management practices and disease history, of which 60 provided both faecal samples and completed questionnaires. Data collected were entered into a custom Microsoft Access database (Microsoft Corporation, USA). Faecal samples were collected across each poultry unit following a ‘W’ pathway, collecting one fresh dropping every two to five paces depending on the size of the unit into 50 ml polypropylene conical tubes, each with a screw top. Samples were fixed in a locally purchased bleach or methylated spirit solution, or 2% (w/v) FIXANAL potassium dichromate solution (Sigma Aldrich, USA). Three to five tubes were filled per unit. Each tube was then properly capped and the contents were thoroughly mixed by vigorous shaking. The samples thus collected were refrigerated at 4°C prior to transportation to UK under IAPO permit for further processing.

### Isolation and identification of oocysts

Faecal samples were homogenised by vigorous vortex mixing for ∼1 min. Initially, 1 ml of each faecal sample was added to 9 ml saturated sodium chloride solution (1∶10 dilution) for oocyst per gram (OPG) quantification using a standard McMaster technique [25]. Subsequently, additional oocysts were then isolated from each sample found to be positive using a modified salt flotation technique. In brief, approximately 50 ml saturated salt solution and 1.6 g NaCl were mixed with 6 ml of faecal homogenate. Two ml of distilled water was added to the top and samples were allowed to separate for 10 minutes before centrifugation (800 g, 9 min). To purify oocysts, the top layer was transferred using a sterile disposable pastette to microcentrifuge tubes, diluted in distilled water 1∶2 (v/v) and centrifuged (10,000 g; 1 min). The supernatant was discarded. Purified oocysts were disrupted in a Mini-BeadBeater-8 (BioSpec, USA) with sterile 0.4–0.6 mm glass beads in sterile phosphate buffered saline and genomic DNA was extracted from the homogenate using a QIAamp DNA Stool Mini kit (QIAGEN, Germany).

### Identification of *Eimeria* species

The 5S ribosomal RNA sequence in the family Eimeriidae is highly conserved across species and present in multiple copies per genome. DNA extracted from samples positive for oocysts by microscopy were initially PCR-amplified for the 5S rDNA sequence to confirm the presence of *Eimeria* parasite DNA. Briefly, each reaction contained 25 ng template DNA, 20 pmol *Eimeria* 5S primers [26], 0.5 U *Taq* polymerase (Invitrogen), 10 mM Tris-HCl, 1.5 mM MgCl_2_, 50 mM KCl and 0.2 mM dNTPs. Standard cycle parameters were 1× (5 min at 94°C), 30× (30 sec at 94°C, 30 sec at 56°C, 1 min at 72°C) and 1× (10 min at 72°C). Samples positive for *Eimeria* 5S rDNA were screened for the presence or absence of each of the seven *Eimeria* species using a validated panel of diagnostic quantitative PCR assays in a non-quantitative survey (PCR as described above, primers as described elsewhere) [27]. All PCR products were visualized on agarose gels and compared against known reference (positive control) parasite DNA samples. No template negative controls included molecular grade water in place of purified genomic DNA. Amplicons were recovered from 5% of all positive PCR reactions using a Qiagen PCR purification kit, cloned using pGEM®-T Easy (Promega, USA) in XL1-Blue *Escherichia coli* (Agilent, USA) and miniprepped (Qiagen, Germany) as described by the respective manufacturers prior to capillary sequencing using T7 plasmid specific primers (GATC Biotech, Germany) to confirm identity.

Subsequently, fragments representing the partial 18S rDNA, internal transcribed spacer (ITS)-1, 5.8S rDNA, ITS-2 and partial 28S rDNA were amplified from a subset of 17 samples (Ghana: 5, Tanzania: 8 and Zambia: 4) using universal ITS-1/-2 primers and PCR conditions as described elsewhere [8]. Each PCR reaction was purified using a Qiagen PCR purification kit, cloned using Strataclone vector pSC-A-amp/kan in Solopack competent cells (Agilent, USA) and miniprepped (Qiagen, Germany) as described by the respective manufacturers prior to capillary sequencing (GATC Biotech, Germany) using T3 and T7 plasmid specific primers (Eurofins MWG Operon, Germany) including 3–6 clones per sample. Sequences were analysed, aligned and compared with publically available sequences in GenBank using CLC Main Workbench (version 5.7.1). Sequences generated here and identified as homologous to OTU-X and OTU-Z ITS-2 [EMBL: HE997165-HE997168] were extracted and aligned with all publically available associated sequences (OTU-X: AM922249-52, sequences presented in Figure S1; OTU-Z: AM922256-8, Figure S2), supporting design of putatively OTU-X and OTU-Z specific primer pairs (OTU-Xfor: 5′-GTGGTGTCGTCTGCGCGT-3′; OTU-Xrev: 5′-ACCACCGTATCTCTTTCGTGA-3′; OTU-Zfor: 5′-TATAGTTTCTTTTGCGCGTTGC′3′; OTU-Zrev: 5′-CATATCTCTTTCATGAACGAAAGG-3′). Primers targeting OTU-Y were designed using published OTU-Y ITS-2 sequences AM922253-5 (OTU-Yfor: 5′- CAAGAAGTACACTACCACAGCATG-3′; OTU-Yrev: 5′- ACTGATTTCAGGTCTAAAACGAAT-3′). All samples were then screened for OTU-X, -Y and -Z genotype parasites by PCR using the conditions described above with the annealing temperature adjusted to 58°C.

### Calculation of profitability

Farm production parameters and costs were used to calculate the outputs, variable costs and fixed costs of individual farms [28]. Briefly, outputs included the cost of bird purchase, the sales of birds (broilers or spent hens), the sale of eggs and the sale of manure as fertilizer. Variable costs included feed, litter, contract labour, transport, disinfectants veterinary costs and pharmaceutical products. Fixed costs included housing, feeders and drinkers, cages, maintenance, water, electricity and interest payments. All monetary amounts were converted to US dollars using local exchange rates available at the time of sample collection. Egg production on layer farms was estimated using data collected on the bird ages and production levels at the start, peak, decline and end of egg production [29]. The gross margin was calculated as *gross margin  =  outputs – variable costs* and the enterprise budget was calculated as *enterprise budget  =  outputs – variable costs – fixed costs* [28].

Missing data were supplemented with regional averages for farms of similar sizes. Calculated gross margins were adjusted by the length of the production cycle and the number of birds per cycle to yield the gross margin per bird per year. Farms were classified as highly profitable if the gross margin was equal to or above the median for each country and production type and lowly profitable if the gross margin was below the median for each country and production type.

**Figure 1:**
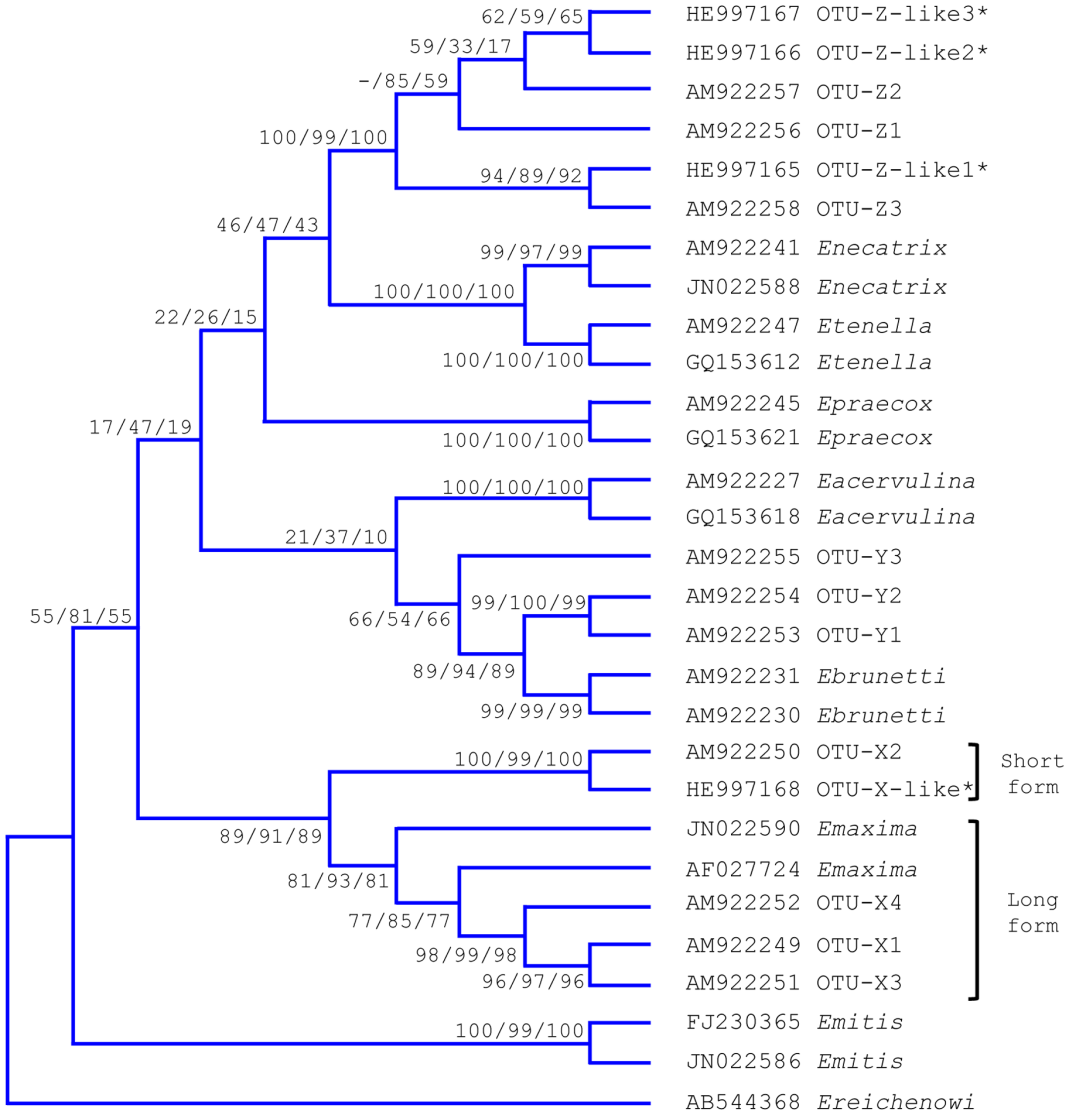
Phylogeny of *Eimeria* species internal transcribed spacer 2 (ITS-2) sequences. Phylogeny produced using Maximum Likelihood (ML), Neighbour Joining (NJ) and Maximum Parsimony (MP) algorithms with *Eimeria* species ITS-2 sequences generated here and publically available (GenBank accession numbers as shown, sequences generated here are indicated with an asterisk ‘*’). Bootstrap values shown for ML/NJ/MP respectively, n = 1000 for each.

Farms were classified by the production type and pathogenicity of the *Eimeria* species present [30]. In the absence of published data defining the OTU parasites their likely pathogenicity was predicted based upon phylogenetic similarity to *Eimeria* species of known pathogenicity, creating a scale of 1–4 (Table 2). Phylogenetic similarity was assessed using Tamura-Nei model Maximum Likelihood, Kimura-2 parameter model Neighbour-Joining and Maximum Parsimony phylogeny using default parameters with 1000 bootstrap replication, constructed in Mega5.1 using publically available and new ITS-2 sequences as shown in Figure 1 and Figures S1–S2; [31]. Thus, OTU-X, identified as a genetic *E. maxima* variant, was classified with this species in pathogenicity group 2 [7], while OTU-Y and OTU-Z were considered to be more pathogenic given closest similarity to *E. brunetti* (group 3) and *E. tenella*/*E. necatrix* respectively (group 4) [8] (Figure 1, Table 2). Where multiple species were present, farms were classified by the most pathogenic species present. Species present were further classified as causing either malabsorptive or haemorrhagic disease (Table 2).

**Table 2:** Pathogenicity and disease type of *Eimeria* species.

Pathogenicity (Group number)	Species	Disease type
Low (1)	*E. mitis E. praecox*	Malabsorptive disease
Medium (2)	*E. maxima E. acervulina* OTU-X*	Malabsorptive disease
High (3)	*E. tenella E. brunette* OTU-Y*	Haemorrhagic disease
Very high (4)	*E. necatrix* OTU-Z*	Haemorrhagic disease

Predicted by phylogenetic comparison with the most closely related species as shown in Figure 1.

### Statistical analysis

Statistical analyses of data were performed using SPSS Statistics version 19 (IBM Corporation, USA). The levels of association between farm profitability, parasite presence and pathogenicity, number of species present, country and production type were investigated using univariable analysis (chi-squared test and Kruskall- Wallis test).

## Results

### Oocysts detected

Faecal samples were collected from 91% (73/80) of farms visited; the remaining farms were excluded as they were in between batches (i.e. no chickens were available to be sampled). Oocysts were detected on 86% (63/73) farms sampled, including 100% (18/18) of farms in Ghana, 94% (15/16) of farms in Tanzania and 77% (30/39) of farms in Zambia (Table 3).

**Table 3:** The number of farms positive for *Eimeria* species and operational taxonomic unit (OTU) sequences on small-scale farms sampled (percentage positive shown in parentheses).

	Ghana			Tanzania			Zambia			
Species*	Total [18]	Broiler [9]	Layer [9]	Total [16]	Broiler [8]	Layer [8]	Total [39]	Broiler [12]	Layer [20]	Village [7]
*E. acervulina*	12 (66.7)	6 (66.7)	6 (66.7)	9 (56.3)	3 (37.5)	6 (75.0)	19 (48.7)	7 (58.3)	10 (50.0)	2 (28.6)
*E. brunetti*	1 (5.6)	0 (0.0)	1 (11.1)	2 (12.5)	2 (25.0)	0 (0.0)	1 (2.6)	0 (0.0)	1 (5.0)	0 (0.0)
*E. maxima*	2 (11.1)	1 (11.1)	1 (11.1)	4 (25.0)	4 (50.0)	0 (0.0)	3 (7.7)	0 (0.0)	2 (10.0)	1 (14.3)
*E. mitis*	7 (38.9)	3 (33.3)	4 (44.4)	8 (50.0)	2 (25.0)	6 (75.0)	2 (5.1)	0 (0.0)	2 (10.0)	0 (0.0)
*E. necatrix*	3 (16.7)	2 (22.2)	1 (11.1)	4 (25.0)	1 (12.5)	3 (37.5)	8 (20.5)	0 (0.0)	8 (40.0)	0 (0.0)
*E. praecox*	6 (33.3)	5 (55.6)	1 (11.1)	7 (43.8)	4 (50.0)	3 (37.5)	8 (20.5)	1 (8.3)	6 (30.0)	1 (14.3)
*E. tenella*	7 (38.9)	3 (33.3)	4 (44.4)	3 (18.8)	2 (25.0)	1 (12.5)	8 (20.5)	3 (25.0)	3 (15.0)	2 (28.6)
OTU-X like	3 (16.7)	1 (11.1)	2 (22.2)	3 (18.8)	2 (25.0)	1 (12.5)	4 (10.3)	1 (8.3)	2 (10.0)	1 (14.3)
OTU-Y like	0 (0.0)	0 (0.0)	0 (0.0)	0 (0.0)	0 (0.0)	0 (0.0)	0 (0.0)	0 (0.0)	0 (0.0)	0 (0.0)
OTU-Z like	2 (11.1)	1 (11.1)	1 (11.1)	3 (18.8)	1 (12.5)	2 (25.0)	4 (10.3)	1 (8.3)	3 (15.0)	0 (0.0)
Any *Eimeria*	18 (100.0)	9 (100.0)	9 (100.0)	15 (93.8)	7 (87.5)	8 (100.0)	30 (76.9)	8 (66.7)	18 (90.0)	4 (57.1)

Percentages do not add to 100% due to presence of multiple species on farms.

**Figure 2:**
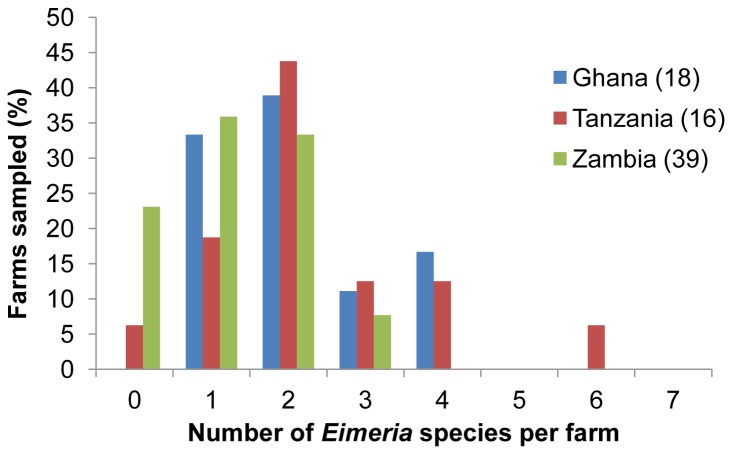
Number of *Eimeria* species identified per farm per sampled country. Chart demonstrating the number of farms in Ghana, Tanzania and Zambia found to host one or more *Eimeria* species parasites. The number presented in brackets indicates the number of farms sampled.

Multiple species were identified on 63% (40/63) of the farms positive for *Eimeria*, with up to six species detected on a single farm (Figure 2). Species complexity was not associated with country or production type. The highly pathogenic species *E. necatrix* was detected on 21% (15/73) of the farms surveyed, all of which were infected by multiple species. Additionally, OTU-Z-like sequences were identified on 12% of farms surveyed including 47% (7/15) of farms infected by *E. necatrix*.

### Farm management practices and disease

Number of *Eimeria* species and pathogenicity level present was not associated with country (chi square, p = 0.560; p = 0.266) or farm type (chi square, p = 0.350; p = 0.334). No farmers reported vaccinating against coccidiosis and very few (13%; 9/67) reported treating with anticoccidial drugs. Use of anticoccidial drugs was not associated with the number of the parasite species (chi square, p = 0.148) or pathogenicity level (chi square, p = 0.279).

### Farm profitability

Economic data were collected for 67 farms. Gross margin and enterprise budgets per bird differed depending on country and production type (Table 4). Farmers were unable to provide exact data on egg production. As records were not available in many farms, this indicates a lack of knowledge of production parameters from many of the farmers.

**Table 4:** Average farm gross margins and enterprise budgets by country sampled.

Country	Farm type	Gross margin per bird per year (median, interquartile range)	Enterprise budget per bird per year (median, interquartile range)
Ghana	Broiler	$11.99 ($3.98, $15.63)	$11.99 ($3.09, $14.01)
	Layer	$6.16 ($4.11, $11.23)	$6.11 ($4.07, $11.17)
Tanzania	Broiler	$2.38 ($0.43, $3.75)	$1.26 ($0.09, $1.78)
	Layer	$8.65 ($5.70, $11.89)	$8.38 ($2.96, $11.28)
Zambia	Broiler	$25.31 ($13.43, $30.25)	$19.15 (−$0.78, $25.70)
	Layer	−$2.49 (−$10.15, $8.41)	−$3.23 (−$11.18, $7.85)

**Figure 3:**
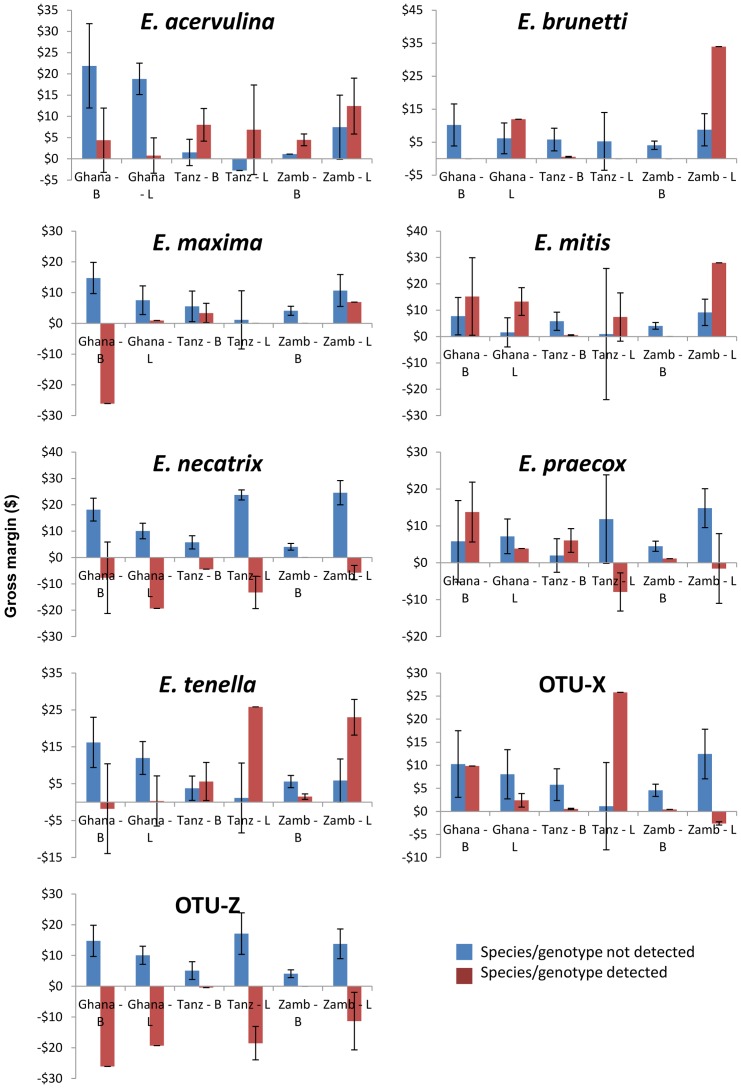
Association of *Eimeria* detection with economic performance of African small-scale commercial poultry producers. The economic productivity of small-scale commercial poultry farms in Ghana, Tanzania (Tanz) and Zambia (Zamb), calculated as gross margin per bird per year ($) for broiler and layer farms (B and L respectively) and categorized by the qualitative detection of individual *Eimeria* species parasites. OTU-Y is not included as no sequences of this genotype were detected.

Pathogenicity of the *Eimeria* species detected was indicative of farm profitability (Kruskal Wallis test, p = 0.02). Farms with parasite species detected that were considered to be very pathogenic (Group 4) were significantly more likely to have low profits (chi square, p = 0.01), as illustrated in Figure 3.

## Discussion

The detection of *Eimeria* in the majority of farms in Ghana, Tanzania and Zambia is a preliminary indication that coccidiosis is widely distributed within small-scale commercial poultry farms in Africa. Although the sample size was limited, the farm level occurrence of *Eimeria* detected in this study was higher than any previously reported study in the test region [13], [14], [15], [16], [17], [18], [19], [20], [21], [22], [23]. Possible explanations include the semi-intensive management practices on these types of small-scale farms. In contrast to traditional backyard poultry, these farms create an environment with high stocking density, commonly populated with specialised, genetically homogenous breeds that may favour disease transmission. Reduced biosecurity practices compared to large industrial farms may exacerbate the problem. Reliance on imported day old chicks can introduce different *Eimeria* species and strains into these environments, as exemplified by the presence of OTUs previously only identified in Australia. The detection of all seven *Eimeria* species known to infect the chicken, with multiple species identified on the majority of positive farms, indicates a complex species population structure. Significantly *E. necatrix*, the most pathogenic of the recognised *Eimeria* species that infect the chicken, was widespread and present in all countries sampled. Many available vaccines, such as Coccivac-B and Paracox-5, do not include *E. necatrix* due to the low prevalence in Europe and North America [32], [33]. Thus, the use of these vaccines designed to control just three or four of the seven species in enclosed, high health settings is inappropriate in this context.

While not yet reported in Europe, Asia, North or South America, the detection of parasites with OTU-X and OTU-Z like genotypes in SSA reveals a much larger range for these genetic variants than has previously been described. The lack of historic samples precludes speculation regarding the geographic origin of these genotypes at this time, but it is important to note that most current *Eimeria*-species specific molecular assays are unlikely to detect these genetic variants. New molecular diagnostics such as has been described here will be required to determine the full range of distribution and explore possible origins.

Farm profitability varied substantially with over a quarter of farms losing money on poultry sales, most notably farms keeping layers in Zambia. This is supported by local observations that many small-scale poultry farms had gone out of business due to rising feed costs and decreasing egg prices. The prices for feed and outputs varied substantially between countries and production systems, affecting the gross margins and enterprise budgets. Similarly, husbandry approaches varied between systems and countries. While these polymorphisms prevented systematic comparison, the use of real-time cost/payment figures provided on farm did allow relative comparison of economic outcome since they reflect actual flock-by-flock data. Using this comparison it was clear that qualitative detection of *Eimeria* presence or absence was a poor indicator of farm profitability. Possible confounding factors include the likely presence of other pathogenic agents, the low proportion of *Eimeria* negative farm comparators and the wide variation in *Eimeria* species-specific pathogenicity. Quantitative detection of specific *Eimeria* species occurrence may have addressed this issue. Nonetheless, qualitative detection of the most pathogenic species *E. necatrix*, with or without the OTU-Z like parasites, did provide a significant indicator for likely farm profitability. While a causal link has not been demonstrated, the presence of highly pathogenic enteric parasites may pose a threat to profitable, sustainable small-scale poultry enterprises in Africa. Inclusion of the OTU sequence-types in these analyses were considered to be essential given the extent of their occurrence, although it must be noted that the pathogenicity attributed to these genotypes are preliminary, based only on phylogenetic proximity to parasites of known pathogenicity. Longitudinal studies are required to determine the effects of infection on economic flock performance and account for temporal changes in infection levels. As the occurrence of coccidiosis has been reported to vary seasonally and local knowledge suggests coccidiosis occurs more frequently during wet seasons, further studies are also required to determine seasonal variations in disease incidence [5], [34].

In conclusion, the risk of coccidiosis is extremely widespread in small-scale commercial farms in Africa and there appears to be a complex parasite population structure. The widespread distribution and genetic variation of *Eimeria* species present in Africa poses a threat to the profitability and sustainability of these enterprises. As many people depend on these production systems for food, work and business viability, coccidiosis has potential implications for food security and poverty alleviation and further research is needed to identify appropriate disease control solutions in these settings.

## Supporting Information

Figure S1 **Clustal-X alignment of operational taxonomic unit (OTU)-X like sequences.** Alignment of OTU-X sequences generated here and publically available (GenBank accession numbers as shown) as used to identify conserved genotype specific sequences for diagnostic PCR primer design.
(TIF)

Figure S2 **Clustal-X alignment of operational taxonomic unit (OTU)-Z like sequences.** Alignment of OTU-Z sequences generated here and publically available (GenBank accession numbers as shown) as used to identify conserved genotype specific sequences for diagnostic PCR primer design.
(TIF)
