# Supplementary figures and images for: Occurrence of Eimeria Species Parasites on Small-Scale Commercial Chicken Farms in Africa and Indication of Economic Profitability
Source: PLoS One. 2013 Dec 31;8(12):e84254. doi: 10.1371/journal.pone.0084254 (PMC3877271; doi:10.1371/journal.pone.0084254)

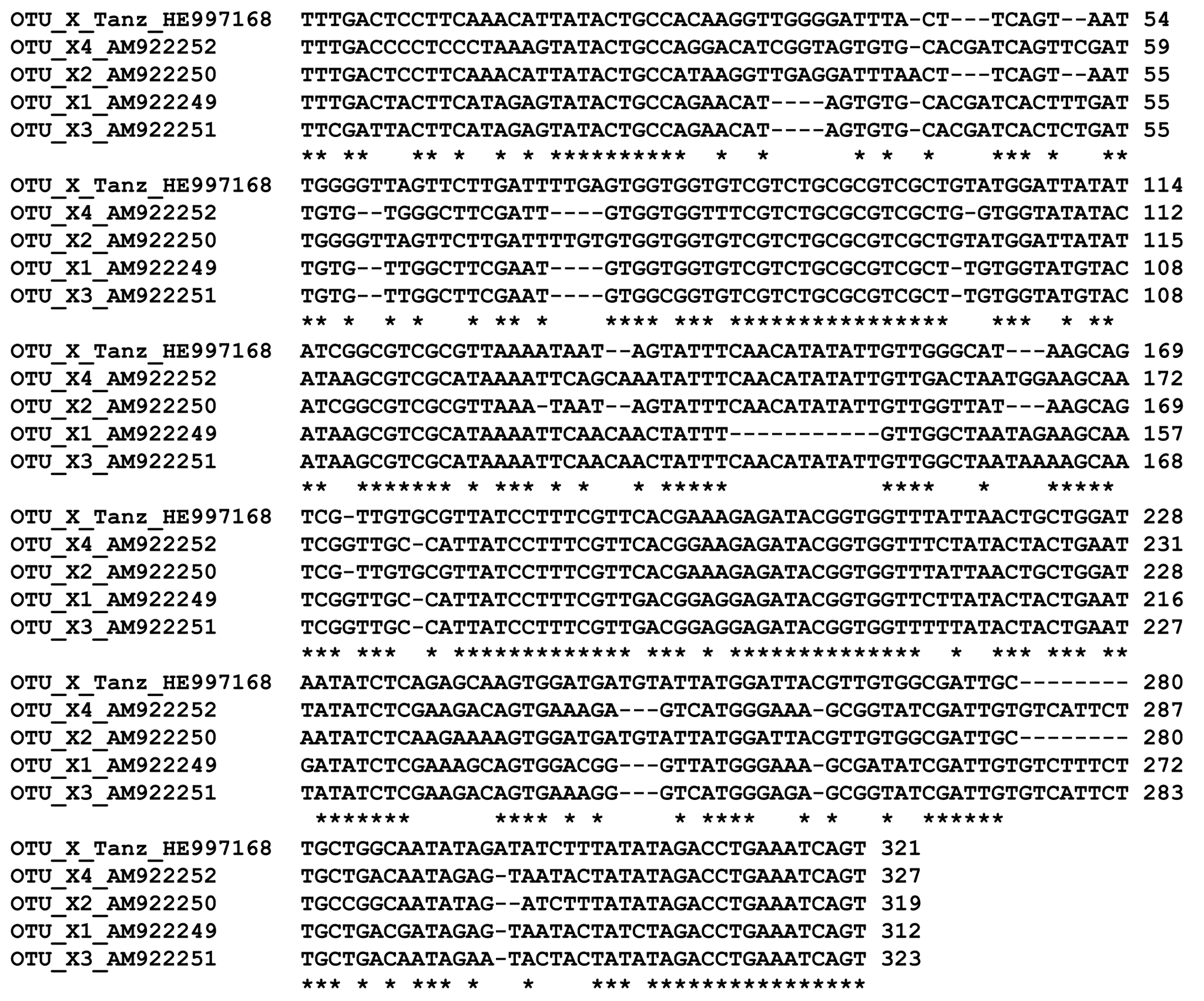

Supplement: Figure S1 — Clustal-X alignment of operational taxonomic unit (OTU)-X like sequences. Alignment of OTU-X sequences generated here and publically available (GenBank accession numbers as shown) as used to identify conserved genotype specific sequences for diagnostic PCR primer design. (TIF) [file pone.0084254.s001.tif]

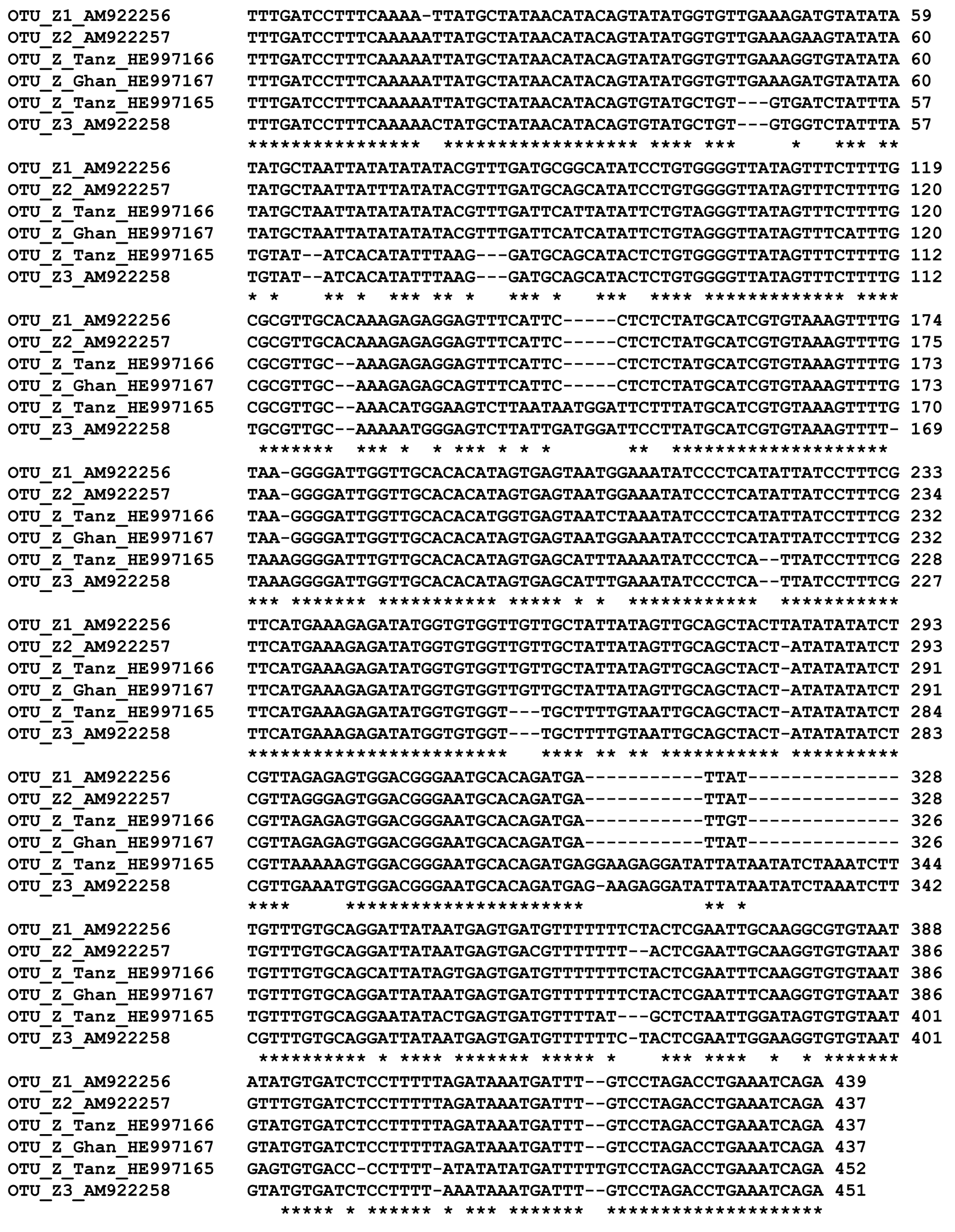

Supplement: Figure S2 — Clustal-X alignment of operational taxonomic unit (OTU)-Z like sequences. Alignment of OTU-Z sequences generated here and publically available (GenBank accession numbers as shown) as used to identify conserved genotype specific sequences for diagnostic PCR primer design. (TIF) [file pone.0084254.s002.tif]
